# Prevalence and characterization of methicillin-resistant *Staphylococcus aureus* isolates from healthy university student athletes

**DOI:** 10.1186/s12941-014-0033-5

**Published:** 2014-08-02

**Authors:** Anna E Champion, Thomas A Goodwin, P Gunnar Brolinson, Stephen R Werre, M Renee Prater, Thomas J Inzana

**Affiliations:** 1 Virginia-Maryland Regional College of Veterinary Medicine, Blacksburg, VA, USA; 2 Edward Via Virginia College of Osteopathic Medicine, Blacksburg 24061, VA, USA; 3 Virginia Tech Carilion School of Medicine, Virginia Polytechnic Institute and State University, Life Sciences 1, 970 Washington St. SW, Blacksburg 24061, VA, USA

**Keywords:** MRSA, Methicillin-resistant, Staphylococcus aureus, Student athletes, SCCmec type, Spa type, MLST, Pulsed field gel electrophoresis, Multilocus PCR

## Abstract

**Background:**

The prevalence of methicillin-resistant *Staphylococcus aureus* (MRSA) has been increasing in the general population, and there is concern that close or physical contact, such as in professional and collegiate sports, may increase spread of MRSA. We sought to determine the prevalence of MRSA colonization of male and female athletes from 9 different sports at a major, Division I University during a 12-week period, and determine the USA and SCC*mec* type from select isolates.

**Methods:**

Swabs for culture of MRSA were obtained from nasal, axillary, and inguinal sites from healthy, asymptomatic student athletes and support staff each week for 12 weeks. Select MRSA isolates were typed by pulsed field gel electrophoresis (PFGE), and the genes encoding for MecA, cassette chromosome recombinase (Ccr), and several toxins were determined by multiplex polymerase chain reaction (PCR). Discrepant results were clarified by multi-locus sequence typing (MLST) and *spa* typing.

**Results:**

Thirty-five percent (78/223) of test subjects were positive for MRSA during the study period, resulting in isolation of 139 MRSA isolates. However, 47% (37/78) of MRSA-positive participants carried MRSA in axillary or inguinal sites, but not in the anterior nares. There was significant correlation between MRSA carriage and participation in wrestling (76%, 19/25; adjusted odds ratio 29.7, 95% CI 5.8-151.5) and baseball (44%, 17/39; adjusted odds ratio 4.4, 95% CI 1.1- 17.4), compared with a staff prevalence of 18.1% (4/22), but other factors were not examined. Multiplex PCR analysis indicated that of the 32 isolates examined 26 could be typed, and all of these carried the SCC*mec* type IV cassette. PFGE typing identified USA types 300, 400, 500, 700, and 800. However, one isolate was not a known USA type, but was identified as a novel ST951 by MLST, and as *spa* type t216. Of the strains typed from the same individual, there was consistency, but also variation and alternation of the SCC*mec* and *spa* types isolated from individual subjects. Various staphylococcal toxin genes were identified in 31 of the 32 isolates analyzed.

**Conclusions:**

Colonization by MRSA was greater in some student athletes than the average carriage rate for the general population, and only 53% of MRSA carriers were identified by nasal cultures. Carriage of MRSA clones on the same individual and transmission to contacts could vary over time, indicating colonization can be a dynamic process that may be difficult to control.

## Background

Methicillin-resistant *Staphylococcus aureus* (MRSA) was first recognized in the early 1960’s as the causative agent of some hospital-acquired (HA) infections, and now accounts for the majority of HA infections in the United States [1],[2]. Community-acquired (CA)-MRSA can be defined as a methicillin-resistant isolate acquired outside of a hospital setting, was acquired within 2 days of hospital admission, or was isolated from a person who has not been hospitalized within 2 years prior to the date of MRSA isolation [3]. CA-MRSA was first recognized in the early 1990’s in Western Australia [4]. By the late 1990’s CA-MRSA [3] had spread worldwide [5], gaining prominence after the death of 4 young children from necrotizing pneumonia who did not have underlying risk factors [6]. CA-MRSA has since become recognized as a major public health concern in the United States, and there is concern it might reach epidemic proportions [7]. Hypervirulence and/or high rates of transmission are trademarks of CA-MRSA, with the ability to cause disease in otherwise healthy individuals. CA-MRSA can cause infections similar to that of HA-MRSA, such as soft tissue and skin infections, often with abscess formation. However, serious life threatening systemic infections, such as necrotizing pneumonia, necrotizing fasciitis, bloodstream infection, and septic shock can also result from CA-MRSA [8],[9].

There have been multiple reports of CA-MRSA infections on college and high school campuses, with a concentration of cases occurring among student athletes [10]. Epidemiological studies have shown that coaching staff and athletes in contact and non-contact sports, as well as spectators, have been implicated as carriers during outbreaks of CA-MRSA [11]. The *mecA* gene, which encodes for the modified penicillin-binding protein 2a (PBP2a) [12], is primarily responsible for methicillin resistance in *S. aureus*. The *mecA* gene is carried on a mobile genetic element, named the staphylococcal cassette chromosome *mec* (SCC*mec*), which has integrated into the *S. aureus* genome [13]. At this time, at least 11 types of SCC*mec* elements (I-XI) have been identified [14]. These SCC*mec* elements share similar characteristics, and contain a *ccr* (cassette chromosome recombinase), which is responsible for site-specific insertion and excision of SSC*mec* into the *S. aureus* genome at the 3’ end of the open reading frame (*orf*X) [15]. SCC*mec* elements are differentiated based on combinations of the types of *mec* and *ccr* genes that have been identified thus far. The two smallest SCC*mec* elements are SCC*mec* IV and SCC*mec* V, which are primarily associated with CA-MRSA. The majority of CA-MRSA strains carry a type 2 *ccr*-class B *mec* complex in a SCC*mec* IV cassette [16]. The SCC*mec* IV cassette is small compared to the SCC*mec* cassettes found in HA-MRSA strains, and often lacks other antibiotic resistance genes besides *mecA*[16]. Okuma *et al.*[16] hypothesized that the smaller size SCC*mec* IV requires a lower cost of fitness than the larger, multi-drug resistant SCC*mec* (normally found in HA isolates), and as a result would favor acquisition and retention of the cassette into the genome. CA-MRSA strains are also more likely to carry toxin genes, including Panton-Valentine leukotoxin (*pvl*), staphylococcal enterotoxins (*sea, seb, sec, sed*, and *see*), toxic shock staphylococcal toxin-1 (tst), and leukocidins (*lukF-PV* and *lukS-PV* [*lukFS-PV*]), than HA-MRSA strains. However, not all CA-MRSA strains produce these toxins [17]–[19].

Chambers *et al*. [20] specifically examined the rates of nasal carriage of MRSA among asymptomatic athletes and coaching staff of infected individuals, and concluded there was little benefit to mass screening. However, those authors suggested that surveillance of multiple sites might improve the detection rates for colonization of asymptomatic carriers due to the presence of distinct flora on other areas of the body. The aim of this study was to assess the prevalence of MRSA colonization among athletes attending a major Division I University and their support staff, characterize the molecular basis of methicillin resistance in some of these isolates, and determine if isolates were exchanged between athletes. The persistence or variance of a genotyped strain on a subject and within a sport throughout the course of a 12-week study was determined. We hypothesized that participants in high contact sports or who experienced repeated exposure to commonly used fomites (e.g. sports equipment) would have a higher incidence of CA-MRSA than participants in low contact sports and who used primarily their own equipment. The results of this research should be noted by coaches, athletic trainers, and sports physicians to make them aware of the higher potential for MRSA infection by some athletes, but to also note that MRSA carriage is not restricted to the nares and does not necessarily result in infection.

## Methods

### Specimen collection

Two hundred twenty-three participants were included in the study, which included predominately college-age athletes, but also 22 of the training and medical sports staff. Athletes and staff with a prior history of MRSA infection (within 12 months) were included and documented as such. Two students reported previous MRSA infections and the course of treatment was noted. All participants were active members of a sports team during the 2007-2008 winter/spring season unless otherwise stated and healthy with no indication of skin or other infections at the start of the study. Whether they lived on or off campus was noted, but not ethnicity, pets, smoking, and whether roommates or family had a previous history of MRSA infection. Every member of the team was swabbed except for twenty-one athletes (25.8% wrestling (WTK), 26.3% baseball (BSB), 12.5% men’s track (MTK), 3.2% women’s Lacrosse (LAX) who refused consent and were excluded. There were no incentives (see Limitations and future work). Specimens from the anterior nares, axillary, and inguinal regions were obtained for culture from each individual with the BBL CultureSwab™ (BD Diagnostics, Sparks, MD) at exactly weekly intervals for 12 weeks, as per the manufacturer’s protocol, in the physician’s Sports Medicine office suite. After collection, specimens were immediately transported to the laboratory for inoculation onto culture medium. This study was approved by the university Institutional Review Board and was carried out exactly as described in the approved protocol (number 2007/041).

### MRSA isolation

**Figure 1:**
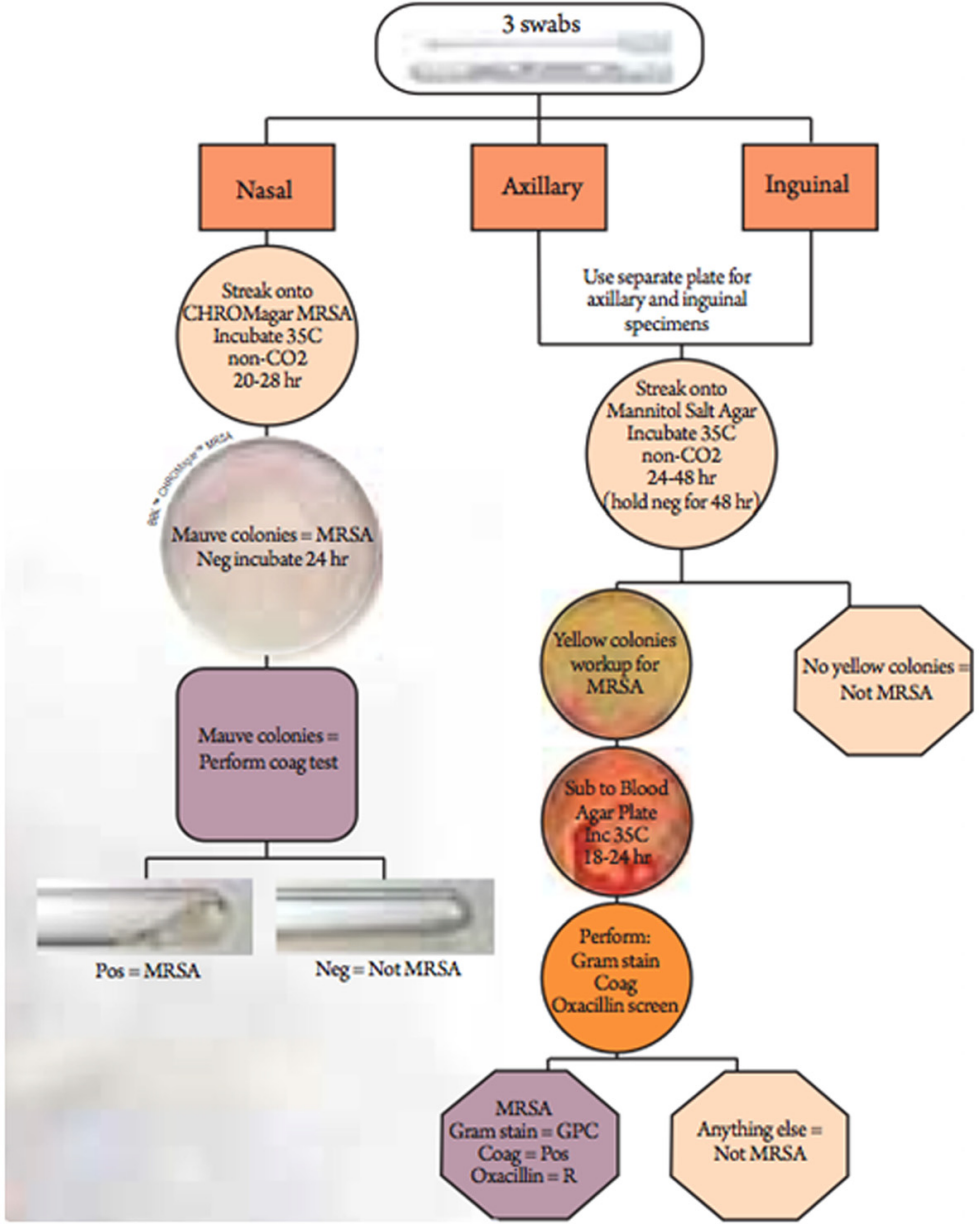
Flowchart of isolate collection and MRSA isolation.

Swabs of the anterior nares were streaked directly onto BBL™ CHROMagar™ MRSA plates (BD Diagnostics, Sparks, MD), which allowed positive confirmation if colonies were mauve at 24 h and presumptive identification if colonies became mauve by 48 h. At 48 h, mauve colonies were collected and confirmed as *S. aureus* by the BBL Coagulase Plasma test (BD Diagnostics). At the time of study CHROMagar™ MRSA plates were approved for identification of MRSA only from nasal swabs. Therefore, standard MRSA isolation techniques were used for axillary and inguinal swabs. Both methods have a greater than 95% specificity for identification of MRSA-positive colonies [21]. Swabs sampled from the axillary and inguinal regions were streaked onto BBL Mannitol Salt Agar (BD Diagnostics) and incubated for 24-48 h. Yellow colonies were subcultured onto Trypticase™ Soy Agar with 5% Sheep Blood (TSA II) (BD Diagnostics) and incubated at 35°C for 18-24 h. Following incubation on TSA II, colonies were Gram-stained and tested for coagulase production. Coagulase-positive strains were then tested for resistance to oxacillin using BBL Oxacillin Screen Agar (BD Diagnostics), and oxacillin-resistant colonies were recorded as MRSA. The overall procedure for identifying MRSA from each type of culture swab is shown in Figure 1. When necessary, confirmation of oxacillin resistance was confirmed by minimum inhibitory concentration (Sensititre, TREK Diagnostic Systems, Cleveland, OH), following approved protocols (M07-A8 and M100-S22) described by the Clinical Laboratory Standards Institute (Wayne, PA). For the purposes of this study, an individual who was “colonized” was defined as having 1-2 positive MRSA cultures over the 12-week period, while an individual who had 3 or more positive cultures during that time period was defined as a “persistent carrier” [22].

### Strain classification using pulse-field gel electrophoresis (PFGE)

PFGE was performed as described by McDougal *et al*. [23]. Briefly, MRSA isolates were suspended in PIV buffer (1 M NaCl, 10 mM Tris- HCl, pH 7.5) to an optical density of 1.2-1.7 at 580 nm, and embedded in 1.6% InCert agarose gel plugs (Lonza Rockland, Inc. Rockland, ME). The cells were lysed in the plugs with lysozyme, and the cell debris removed before enzyme digestion with *Sma*I. The plugs were loaded onto a 1.2% agarose gel for PFGE using a CHEF-DR II apparatus (Bio-Rad, Hercules, CA). Electrophoresis parameters were 200 V (6 V/cm), temperature 4°C, initial time 5 sec, final time 40 sec, and the total run time was 20 hr. Gels were stained using 1.5 μl/ml of ethidium bromide. PFGE types were assigned by matching the banding patterns of isolates to those of the CHEF lambda DNA standard (Bio-Rad, 1703635) and to control strains of known USA type. Photographed gel profiles were analyzed with BioNumerics software (Applied Maths, Kortrijk, Belgium) in comparison to established profiles of USA type strains [24].

### SCC*mec* genotyping by PCR

Approximately 5 bacterial colonies from a single clone were suspended in 100 μl of water and heated at 100°C for 10 minutes to extract DNA. Multiplex PCR was performed as described by Zhang *et al.* for SCC*mec* and *ccr* typing [25]. Eight pairs of primers were used for SCC*mec* types I, II, III, IVa, IVb, IVc, IVd, and V, 2 pairs of primers for the *mec* gene, 4 primers for types 1, 2, or 3 *ccr*, and 1 primer pair for type 5 *ccr*. The primers used were exactly those previously described for amplification of these genes [25]. Amplification was initiated at 94°C for 5 min followed by 10 cycles of 94°C for 45 s, 65°C for 45 s, 72°C for 1.5 min, and then 25 cycles of 94°C for 45 s, 55°C for 45 s, 72°C for 1.5 min, and a final extension at 72°C for 10 min. PCR reactions were performed in triplicate to reduce error.

### Toxin gene identification by PCR

DNA was extracted from approximately 5 bacterial colonies, as described above, and 1 μl of extract containing the DNA was added to each reaction mix. Multiplex PCR was performed as described [26]. Briefly, primer pairs designed for amplification of *sea, seb, sec, sed, see, femA, mecA, eta, etb,* and *tst,* were separated into 2 sets of primer mixes, with *femA* included in mix B, as described [26]. A simplex reaction containing *lukFS-PV* primers (F-5’ TTACACAGTTAAAGAA and R-5’ AATGCAATTGATG) (20 pmol) was used for detection of the *pvl* toxin gene. The amplification protocol was initiated at 94°C for 5 min followed by 35 cycles of 94°C for 2 min, 57°C for 2 min, 72°C for 1 min, and a final extension at 72°C for 10 min. The PCR reactions were performed in duplicate.

### Multi-locus sequence typing (MLST)

An isolate that was untypable by multiplex PCR and PFGE was subjected to MLST using previously published primers and conditions [27]. MLST sequence types were determined through the website, www.mlst.net. A USA 300, *pvl* + strain was used as a control.

### *spa* typing

To confirm results determined by PFGE and SCC*mec* typing, the polymorphic X region of the *spa* gene was amplified using primers *spa*1095F (5′-AGACGATCCTTCGGTGAGC-3′) and *spa*1517R (5′-GCTTTTGCAATGTCATTTACTG-3′) and sequenced as described previously [28]. The sequences were analyzed using Ridom StaphType software version 2.2.1 (Ridom GmbH, Germany).

### Statistical analyses

Data were summarized as the median (range) for age, class year, and number of roommates while frequency tables were generated for the categorical variables that included sport, residence (off campus vs. dorms), previous soft tissue infection, previous MRSA diagnosis, and MRSA carrier status (the primary outcome). The statistical null hypothesis was: there is no association between MRSA carrier status and the hypothesized risk factors for playing a specific sport or other category. The results were tested using univariate and multivariate analyses. For univariate analyses, associations between carrier status and the demographics of age, class year, and number of roommates were tested using the Wilcoxon 2 Sample Test. Associations between carrier status and each type of sport, gender, residence, and previous soft tissue infection were tested using the Pearson Chi-Square Test. The association between carrier status and a previous MRSA diagnosis was tested using the Fisher’s Exact Test. Data on race were not available for statistical analysis. For multivariate analyses, all the hypothesized risk factors (excluding gender) were subjected to stepwise logistic regression modeling to select possible predictors for carrier status. Gender was excluded from multivariate analyses because it was perfectly correlated with the sport. All analyses were performed using SAS version 9.1; Cary, NC, USA.

## Results

### Prevalence of MRSA carriage

Two-hundred twenty-three subjects were enrolled in the study. Overall, 139 total positive cultures were obtained from 78 subjects over the 12-week study period. The prevalence for MRSA carriage was 34.9% (78 out of 223 subjects) (Table 1). Sixty-two of the positive cultures were obtained from the anterior nares, 19 from axillary swabs, and 58 from the inguinal region. Of the 78 positive subjects, 19 harbored MRSA in more than one site, and 59 were positive at only one site (22 nasal, 6 axillary, 31 inguinal). Thus, 37 of the 78 MRSA-positive participants (47%) were not colonized in the anterior nares (data not shown; some isolates are presented in Table 2).

**Table 1:** Prevalence of MRSA by sport in comparison to medical staff

**Sport played**	**#MRSA carriers/total # athletes enrolled per sport**	**percentage colonized per sport/percentage colonized overall**	**Adjusted odds ratio (95%) CI)**^**a**^	**P-value based on comparison to staff**
Men’s wrestling	19/25	76.0/23.8	29.7 (5.8 – 151.5)	<0.0001
Men’s baseball	17/39avb	43.6/21.0	4.4 (1.1 – 17.4)	0.036
Women’s tennis	4/7	57.1/4.9	4.2 (0.6) – 29.4)	0.143
Women’s softball	8/19	42.1/9.9	4.1 (0.9 – 19.0)	0.069
Men’s tennis	3/10	30.0/3.7	3.8 (0.6 – 22.0)	0.140
Men’s track	11/29	37.9/13.6	3.5 (0.8 – 14.6)	0.091
Women’s basketball	1/12	8.3/1.25	N/A^b^	N/A^b^
Women’s track	7/28	25.0/4.9	2.0 (0.4 – 8.9)	0.371
Women’s lacrosse	4/32	12.5/4.9	0.8 (0.2 – 4.2)	0.832
Staff	4/22	18.1/1.8	1 (reference group)	-

^a^Homer and Lemeshow Goodness-of-Fit Chi-Square for the multivariate model was 0.000 (P = 1.000) indicating a good fit.

^b^Excluded from the multivariate model because only 1 participant has a positive result for MRSA.

**Table 2:** Molecular characteristics of 32 MRSA isolates carried by winter/spring athletes at a Division I University

**Isolate source**				**PFGE type**^**a**^	***spa*** **type**		**Multiplex typing PCR**^**b**^	
**Sport**^**c**^	**Participant #**	**Week**	**Sampling site**^**d**^			***ccr*-*mec***	**SCC** ***mec*** **Type**	**Toxin gene profile**
WR	35	5	I	USA700	n/d	2-B	IVb	*eta, lukFS-PV*
WR	41	6	I	USA700	n/d	2-B	IVb	*eta, lukFS-PV*
WR	41	10	I	USA700	n/d	2-B	IVb	*eta, lukFS-PV*
WR	47	3	A	Unknown^e^	t216	2-B	Undetermined^f^	*seb, sed, etb, tst, lukFS-PV*
WR	52	5	I	USA500	txAA	2-B	IVb	*sea, see, eta, lukFS-PV*
WR	52	6	I	USA700	n/d	2-B	IVb	*eta*
WR	55	2	N	USA300	t008	2-B	IVd	none detected
WR	55	3	N	USA300	t008	2-B	IV	*lukFS-PV*
WR	55	3	A	USA300	t008	2-B	IVa	*etb, tst, lukFS-PV*
WR	55	5	N	USA300	t008	2-B	IV	*lukFS-PV*
WR	55	7	I	USA300	t008	2-B	IV	*sec, etb, tst, lukFS-PV*
WR	55	7	N	USA300	t008	2-B	Undetermined^f^	*eta, lukFS-PV*
WR	55	8	N	USA300	n/d	2-B	IV	*etb, tst, lukFS-PV*
WR	55	8	I	USA300	t008	2-B	IV	*etb, tst, lukFS-PV*
WR	57	1	N	USA400	t128	2-B	IVa	*sea, see*
WR	57	3	N	USA400	t128	2-B	IVa	*seb, sec, sed, see, etb, tst, lukFS-PV*
WR	57	5	N	USA400	n/d	2-B	IVa	*seb, sec, see, etb, tst, lukFS-PV*
WR	57	8	N	USA400	t128	2-B	IVa	*eta, lukFS-PV*
WR	57	9	N	USA400	n/d	2-B	IVa	*seb, see, eta*
WR	57	12	N	USA400	n/d	2-B	IVa	*seb, see, lukFS-PV*
WR	65	12	N	USA800	t088	2-B	Undetermined^f^	*tst*
WR	81	6	N	USA800	t002	2-B	Undetermined^f^	*lukFS-PV*
SB	86	1	I	USA400	t008	2-B	IVa	*sea, see, eta, lukFS-PV*
SB	90	1	N	USA800	n/d	2-B	IVd	*etb, lukFS-PV*
SB	90	5	I	USA800	t002	2-B	Undetermined^f^	*etb, lukFS-PV*
SB	90	5	N	USA800	t128	2-B	Undetermined^f^	*lukFS-PV*
SB	90	7	N	USA800	n/d	2-B	IVd	*eta, lukFS-PV*
SB	90	8	N	USA800	t002	2-B	IVa	*lukFS-PV*
BSB	108	5	A	USA300	txAB	2-B	IV	*etb, lukFS-PV*
BSB	108	11	I	USA300	t334	2-B	IVd	*lukFS-PV*
BSB	111	1	I	USA300	t008	2-B	IVa	*sec, etb, tst, lukFS-PV*
WBB	234	1	I	USA400	n/d	2-B	IVa	*seb, see, eta*

^a^PFGE patterns were assigned as described [23].

^b^Multiplex PCR for SCC*mec* and *ccr* typing was carried out as described [25], and for toxin gene identification [26].

^c^WR: Wrestling, SB: Softball, BSB: Baseball, WBB: Women’s Basketball.

^d^Sampling site: I, inguinal; A, axillary; N, nasal.

^e^Unknown PFGE pattern; MLST was identified as ST951.

^f^Failed to specifically subtype using primers for SCC*mec* I-IV.

Univariate analysis indicated there was a significant correlation between MRSA colonization, the sport (76.0% [19/25] for men’s wrestling vs. 43.6% [17/39] for men’s baseball vs. 57.1% [4/7] for women’s tennis vs. 42.1% [8/19] for women’s softball vs 30.0% [3/10] for men’s tennis vs. 37.9% [11/29] for men’s track vs. 25.0% [7/28] for women’s track vs. 12.5% [4/32] for women’s lacrosse; p < 0.0001), and gender (48.5% [50/103] for men vs. 24.5% [24/98] for women; p = 0.0004), as well as a previous history of skin or soft-tissue infection (72.2% [13/18] for yes vs. 35.4% [62/175] for no; p = 0.0023). There was no statistically significant association with residence status (36.1% [26/72] for dorms vs 42.6% [49/115] for off-campus; p = 0.377), class year (median was 2 [second year on campus] for both colonized and non-colonized individuals, range 1-4 and 1-5, respectively; p = 0.709), age (median was 20 years for individuals colonized with MRSA vs. 19.0 for individuals not colonized, range for both groups 18-23; p = 0.312), or number of roommates (median was 2.5 for individuals colonized with MRSA vs. 2.0 for individuals not colonized, range for both groups 0-9; p = 0.116). Only 2 participants has a previous history of a MRSA infection, which would not support a meaningful conclusion.

**Figure 2:**
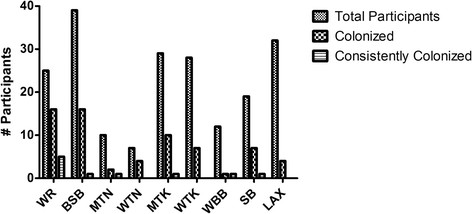
**Prevalence of MRSA colonization in athletes by sport.** Men’s wrestling had the highest percentage of MRSA carriers (19 out of 25 participants or 76.0%). In other sports, 8.3-57.14% of participants carried MRSA. Athletes participating in men’s wrestling (p = 0.0023) and men’s baseball (p = 0.036) were statistically significant for colonization with CA-MSRA. WR: wrestling, BSB: baseball, MTN: men’s tennis, WTN: women’s tennis, WBB: women’s basketball, SB: softball LAX: women’s lacrosse.

Multivariate analysis of the factors analyzed indicated that the sport was the only predictor of MRSA colonization. Table 1 also shows sport-specific classification using the medical staff as the baseline. Compared with a staff prevalence of 18.1% (4/22), the only athletes for which colonization with MSRA was significantly more common than to the comparator group (medical staff) were in men’s wrestling (76%, 19/25; adjusted odds ratio 29.7, 95% CI 5.8 – 151.5; p <0.0001) and men’s baseball (43.6%, 17/39; adjusted odds ratio 4.4, CI 1.1 – 17.4; p = 0.036) (Table 1). When individual sports were compared with the total number of athletes colonized with MRSA, men’s wrestling proved to have the highest percentage of colonized participants (19 out of 25; 76.0%) (Table 1, Figure 2). Women’s basketball had the lowest number of colonized and persistent carrier participants, followed closely by men’s tennis. There were no persistent carrier participants on the women’s lacrosse team (Figure 2). In other sports the sport colonization rates varied from 8.3-57.1%, and the overall colonization rates were 1.25 to 21.0% (Table 1). However, these numbers reflect the percent of participants who had at least one MRSA isolate recovered during the 12-week study. Persistent carriers (subjects with 3 or more MRSA isolates during the 12-week test period), were observed only in men’s wrestling and baseball (see above). The other sport’s persistent carrier rates dropped to 2.5-13.6%, which was not significantly different from the comparator group (Medical Staff, data not shown). The low number of strains carried by more than one person was likely affected by the fact that, on average, approximately one third of participants were unavailable at the weekly collection time. Therefore, 12 samples from each site were not able to be obtained from every participant during the study. Weeks with no collection data for a participant were not included in statistical analyses. Medical staff screening yielded 4 MRSA colonized participants among a total of 22 participants (18.2%).

### PFGE analysis

**Figure 3:**
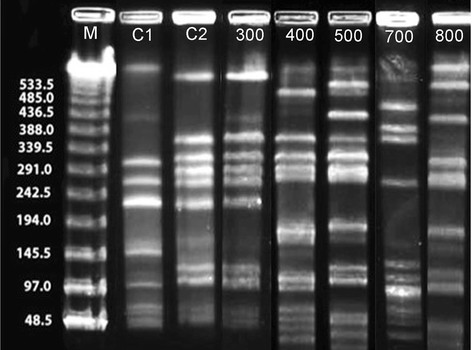
**Examples of*Sma*I-PFGE banding patterns of MRSA isolates.** The PFGE-tenable isolates were categorized into 5 USA types. Lanes 500 and 700, representing USA types USA500 and USA700, were isolated from the inguinal site of the same individual 1 week apart. Lanes: M, molecular size standards; C1, control MRSA strain BAA1685, type USA200; C2, control MRSA strain BAA1556, type USA300; 300, type USA300 isolate; 400, type USA400 isolate; 500, type USA 500 isolate; 700, type USA700 isolate; 800, type USA800 isolate.

Of the 139 MRSA isolates collected, 32 confirmed MRSA isolates were selected for further characterization based on repeated participant colonization or based on participation in a sport with a high persistent carriage rate. The 32 MRSA isolates further tested were from 13 individuals and consisted of five different pulse-field types (PFT): USA300, USA400, USA500, USA700, and USA800 [23] (Table 2 and Figure 3). Of the 13 participants represented in Table 2 from whom MRSA isolates were characterized, 6 were colonized with USA300/400 MRSA types. Of interest was that in this population 3 of the participants were colonized with USA700 MRSA isolates (one participant was also colonized with USA500 [Figure 3]) and 3 were colonized with USA800 MRSA isolates. One isolate (a wrestler’s axillary isolate; 47A-3) was not classified into any of the known USA strain designations after digestion with *SmaI* and PFGE.

### SCC*mec* typing

All of the isolates tested that could clearly be typed harbored SCC*mec* IV (Table 2). Of the 26 isolates confirmed to harbor SCC*mec* IV, subtyping was successful in 20 of these. Eleven were subtype IVa, 5 were subtype IVb, and 4 were subtype IVd. None of the isolates harbored SCC*mec* IVc. Of the isolates tested in this study (Table 2), six isolates (47A-3, 55 N-7, 65 N-12, 81 N-6, 90I-5, and 90 N5) were unable to be classified into a known SCC*mec* type using primers for SCC*mec* I-IV, and IVa-IVd, which is not uncommon [29]. Since the completion of this study several new SCC*mec* types have been discovered indicating the vast genetic diversity of the SCC*mec*, and it is possible these undetermined isolates could be a newer type not tested for. Isolates 90I-5 and 90 N-5 were from the same baseball participant on the same collection date, but from different sites. All isolates tested were of *ccr-mec* type 2-B.

### MLST and *spa* typing

The unclassified isolate 47A-3 was subjected to MLST typing and was identified as a clonal type of ST951. This same isolate was also found to be *spa* type t216. There are no previously published MRSA strains with these two profiles. Isolate 47A-3 could be a novel *S. aureus* strain, and may be the reason it did not fall into a previous classification type by PFGE or SCC*mec* typing. Isolates that exhibited clonal variation within a subject over the 12-week period or those isolates that were untypeable by either previous method were also selected for *spa* typing. Twenty-one isolates were tested, which were classified into 8 *spa* types. Three subjects encompassing 9 of the isolates tested were *spa* type t008. Six of these isolates were from subject 55 and all were of PFGE type USA300. The *spa* types t128 and t002 were each found in the same softball subject, but from two different sites obtained on the same date (nares and inguinal); both strains were USA800 and were of unclear SCC*mec* type. The *spa* types t128 and t002 were also isolated from two different wrestlers. The *spa* types t216, t088, t334, txAB, and txAA (the latter two are novel spa types) were recovered only once, but may have been represented in isolates not tested.

### Detection of toxin genes

Nine staphylococcal toxin genes were detected among the 32 MRSA isolates analyzed (Table 2). The most common were *lukFS-PV* (Panton-Valentine leukocidin) (81%; 26 of 32 isolates; 19% of isolates tested positive for only *lukFS-PV*), and the exfoliative toxins *eta* (34%, 11 of 32 isolates), and *etb* (34%; 11 of 32). Other toxin genes detected included the enterotoxin genes *see* (25%, 8 of 32), *sea* (9%, 3 of 32), *seb* (19%, 6 of 32), *sec* (13%, 4 of 32), and *sed* (6%, 2 of 32). Nine isolates carried the toxic shock toxin gene *tst* (28%). A single nasal isolate (55 N-2) carried by participant 55 had no toxin genes, but other isolates recovered from participant 55 over the 12-week study were positive for the presence of multiple toxin genes.

### Isolate variation over time

Isolates from several participants who were repeatedly colonized with MRSA were characterized in order to identify isolate changes over 12 weeks. Wrestling participant 55 carried one clone: USA300 of *spa* type t0008 for 8 weeks. Wrestling participant 57 was colonized with MRSA for 6 of 12 weeks; all isolates were USA400 clones, *spa* type t128. Five isolates from participant 90 were USA800 isolates, but of those tested, two different *spa* types were identified: t002 and t128. Similarly, two isolates from baseball participant 108 were both of USA300, but of distinct *spa* types. In addition, two different USA types were identified from wrestler #52, 1 week apart, and both were from the inguinal region (Table 2). Throughout the study, isolates were recovered from these participants that had varying toxin gene profiles. Of those harboring isolates with toxin genes, the most prevalent throughout were *seb, see,* and *lukFS-PV*.

Isolate characteristics were also examined between participants within the same sport. Two transiently colonized wrestling participants had the same USA700 clone containing *eta* and *lukFS-PV* toxin genes. Participant 55 was colonized for 8 weeks with a USA300 clone, but different toxin genes were identified in the isolates. Of the 22 MRSA isolates from wrestlers that were genetically characterized, there were 5 different clones (USA300, 400, 500, 700, and 800) and one unidentifiable isolate type. Participant 57 was continuously colonized with a USA400 clone, while participants 65 and 81 were transiently colonized with a USA800 isolate with varying toxin genes (data not shown). The isolates analyzed from baseball participants were all from a USA300 clone that carried varying toxin genes. From our study, it is unclear whether this was one USA300 clone that gained or lost toxin genes throughout the 12 weeks or separate clones of USA300. The genes *lukFS-PV* were the only toxin genes consistently identified from all the isolates from baseball participants. However, because up to one-third of the participants were not available for culture each week, the persistence or variation of isolates from a given athlete were incomplete.

## Discussion

During the past 15 years CA-MRSA isolates have been recovered from otherwise healthy individuals outside of the hospital setting. Infections due to CA-MRSA can be unpredictable, invasive, and more deadly than HA-MRSA. As a result, infections with such isolates must be carefully monitored, and the risks due to carriage and transmission of such isolates require further investigation and understanding. The prevalence of MRSA infections in athletes can be much higher than the general population, particularly in athletes in contact sports [30]. However, there are a lack of data regarding the prevalence of MRSA carriage in healthy college athletes, and the molecular characterization of such isolates. Our study sought to address the prevalence and molecular types of MRSA in this population.

It is well established that the anterior nares serves as a reservoir for *S. aureus*[31]–[33]. Several studies have examined the colonization rates of both symptomatic and asymptomatic athletes and spectators. The largest group studied has been American football players. Nguyen *et al*[30] reported an outbreak of CA-MRSA skin and soft tissue infections in 11 players on a college football team. Cultures of the anterior nares only identified 26 of 99 healthy players carrying *S. aureus,* of which 8% were MRSA. However, only 1 sample was obtained, rather than multiple samples over 12 weeks, as in this study. In our study the overall colonization rate of MRSA in all athletes and their support staff was 36.8%. Begier *et al*. [34] reported an outbreak of MRSA infection among college football players due to a USA300 clone that was positive for the *lukFS-PV* genes. Nasal swabs were collected from the athletes and staff, but none were found to carry MRSA. However, 48.5% were colonized with MSSA. Kazakora *et al.*[11] reported similar findings in regard to an outbreak of MRSA infections in a professional football team. Nasal swab cultures were collected from all athletes, but again MRSA carriage was not found. [34]. It is likely these participants were carrying MRSA, but in sites other than the anterior nares. Although the number of participants per sport was relatively small, and ethnicity, socio-economic background, or family history of MRSA infection was not noted, this study was distinct in that there had been no reported outbreak of MRSA in the population we examined, and only one active infection with MRSA occurred during the 12-week study. A novel aspect of our study was continuous sample collection from 3 anatomic sites over a 12-week period, as opposed to a one-time nares-only collection, as in previous studies [11],[34]. We expected that culturing multiple sites would identify a higher rate of transient MRSA colonization. Transient MRSA isolation was evident in multiple participants throughout the study, either with the same MRSA isolate (e.g. participant #s 41, 55, 57) or with varying isolates (e.g. participant #s 52, 90, 108). However, the profile of persistent or transient carriage of a particular isolate by a participant was incomplete due to a substantial number of participants not being available for culture on any given week.

As reported in previous studies [10],[11], athletes in contact sports have higher carriage rates of MRSA. In fact, participants in sports involving greater physical contact, have the most significant carriage rate for MRSA. In our study, of the parameters examined, the sport was the only predictor of MRSA colonization. The highest colonization rate was associated with the contact sport wrestling, followed by baseball. Athletes participating in football were not tested because only athletes participating in Spring sports were involved in this study. However, the sport with the lowest level of colonization was women’s basketball, and no participants in women’s lacrosse were found to be persistent carriers. Since less contact would be expected in baseball than in basketball or lacrosse (though not to the level of contact in wrestling) gender may also be a factor in CA-MRSA colonization. Nonetheless, these results suggest that athletes who have more physical contact with commonly used fomites or teammates may need to be monitored more closely for signs of potential MRSA infections.

In the United States, MRSA clones USA300, USA400 and often USA700 are the most prevalent and carry the SCC*mec* IV cassette [23]; USA300 is currently responsible for the majority of morbidity and mortality associated with MRSA, particularly in athletes [6],[10]. Of the select isolates represented in Table 2, MRSA carriage among the athletes in this study followed the same pattern; all isolates from which a clear *SCCmec* type could be identified were confirmed to carry a SCC*mec* IV cassette, which is most commonly found in USA300 and USA400 clones. As expected, USA300/400 clones made up over 69% of the confirmed type IV isolates tested. Of the 13 participants from whom MRSA isolates were characterized, 6 were colonized with USA300/400 MRSA types. Of interest was that in this population 3 of the participants were colonized with USA700 MRSA isolates and 3 were colonized with USA800 MRSA isolates. Although strains of USA700 commonly occur in the community, USA800 isolates are more often associated with hospital-acquired infections, but community isolates are not uncommon [23]. Furthermore, Stevens *et al*. reported the presence of USA800 from women’s collegiate basketball players [35]. Unfortunately, we did not determine if any of the participants from whom USA800 isolates were recovered had visited a hospital or were in contact with people who had been hospitalized or had visited a hospital. However, one isolate could not be classified into an established USA type by PFGE or PCR, but was determined to be a novel MLST and *spa* type. This was the only MRSA isolate from this individual, and therefore due to its transient nature its significance cannot be determined. The most common *spa* type found among these isolates was t008, which represents only 5.99% of isolates in the Ridom SpaServer database (http://www.spaserver.ridom.de), but in our study type t008 had a much higher frequency (42.8% of isolates typed and 3 of 13 participants studied). The t008 type corresponds to the common USA300 clone, and all of the USA300 isolates characterized in this study were *spa* type t0008, as well as one USA400 type. The two other *spa* types found in multiple participants were also more common than what is represented in the Ridom database: t002 accounted for 14.2% of our isolates (6.4% average) or in 2 of 13 participants studied. The *spa* type t128 accounted for 19% of our isolates (0.05% average) or in 2 of 13 participants studied (http://www.spaserver.ridom.de).

We were also interested in determining if MRSA isolates from healthy participants carried any of the staphylococcal toxin genes, and if so which ones. We detected 9 toxin genes among the 40 MRSA isolates tested. The most common genes found were *lukFS-PV, eta,* and *etb*. The *lukFS-PV* genes encode for the Panton-Valentine leukocidin toxin. MRSA isolates carrying these genes have been shown to be responsible for more severe disease and clinical symptoms, including necrotic lesions of the skin [36]. The *eta*/*etb* genes encode for exfoliative toxins that may enhance the transmission/progression of a MRSA infection through skin-skin contact, due to the destruction of the epidermal barrier [37]. However, during the course of this study only 1 athlete developed a MRSA infection. Although the presence and expression of toxin genes may potentially make the bacteria more virulent following infection, there was no correlation between the presence of toxin genes and predisposition to infection in these athletes. Therefore, other predisposing conditions may be necessary to initiate infection.

Most participants were not colonized with the same clone continuously throughout the study, nor were they colonized in the same site. While it is quite possible that the presence of MRSA was missed on a particular culture or due to a participant’s occasional absence, the variation in toxin genes further suggested that some participants may be prone to carriage or colonization with MRSA, but that residence by individual clones varies. Thus far, studies are lacking that have followed colonization of healthy individuals over time with MRSA to evaluate the persistence of individual strains on a subject over time. Such results have not been commonly reported because most studies have examined MRSA from infected individuals, rather than the prevalence of carriage and colonization in a healthy population. Based on PFGE analysis, it was apparent that strains were transmitted between teammates participating in wrestling and baseball. The mechanism of such transmission was not determined, but is likely through direct contact (particularly in sports such as wrestling) or indirectly through common contact with contaminated fomites (which may occur in locker rooms or handling common pieces of equipment).

## Conclusions

This study indicated that participants in sports are more likely to carry or be colonized with MRSA than the general population, that such carriage may occur in multiple sites other than the nares, and that transient colonization with different strains was common. Precautions to minimize transmission of MRSA between athletes or equipment and facilities used by athletes and staff may be warranted.

### Limitations and future work

A questionnaire was given to each participant asking about residence, previous history of skin or soft tissue infection within 12 months and any previous history of MRSA diagnosis. The specific participants with previous MRSA infection history were not identified to those processing the samples. Therefore, we know only that 1 baseball player and 1 wrestler were previously infected with MRSA. Comparisons between these previously infected individuals and other athletes are lacking. Due to participants scheduling conflicts, twelve weeks of sample collections were not completed on all participants. Future work could include molecular characterization on all 139 MRSA isolates. This could allow for more in-depth comparisons of isolates shared between sports, or comparison of staff versus sport participants.

## Abbreviations

MRSA: Methicillin-resistant *Staphylococcus aureus*

CA-MRSA: Community-acquired MRSA

HA-MRSA: Hospital-acquired MRSA

PFGE: Pulsed filed gel electrophoresis

PCR: Polymerase chain reaction

MLST: Multi-locus sequence typing

PBP2a: Penicillin binding protein 2a

SCC*mec*: Staphylococcal cassette chromosome mec

*Ccr*: Cassette chromosome recombinase

*Pvl*: Panton-Valentine leukocidin gene

*Tst*: Toxic shock staphylococcal toxin-1

*lukFS-PV*: Panton-Valentine leukocidin

PFT: Pulse field type

## Competing interests

The authors declare that they have no competing interests. Media was provided by BD Diagnostics, Sparks, MD.

## Authors’ contributions

AC carried out cultures and molecular typing and wrote the initial draft and reviewed the final draft of the manuscript. TAG conceived the protocol and collected specimens for processing. PGB conceived the protocol and collected specimens for processing. SW performed the statistical analyses. MRP helped conceive the experiments and protocols. TJI conceived the molecular studies, oversaw sample culturing and processing, and wrote the final draft of the manuscript. All authors read and approved the final manuscript.
